# Evidence that cultural groups differ in their abilities to detect fake accents

**DOI:** 10.1017/ehs.2024.36

**Published:** 2024-11-20

**Authors:** Jonathan R. Goodman, Enrico Crema, Francis Nolan, Emma Cohen, Robert A. Foley

**Affiliations:** 1 Leverhulme Centre for Human Evolutionary Studies, Fitzwilliam St, Cambridge CB2 1QH, UK; 2 Phonetics Laboratory, 5 West Rd, Cambridge CB3 9DP, UK; 3 Social Body Lab, The Pauling Centre, 58a Banbury Rd, Park Town, Oxford OX2 6QS, UK

**Keywords:** Accents, language, mimicry, cheater detection, tag-based cooperation

## Abstract

Previous research in the evolutionary and psychological sciences has suggested that markers or tags of ethnic or group membership may help to solve cooperation and coordination problems. Cheating remains, however, a problem for these views, insofar as it is possible to fake the tag. While evolutionary psychologists have suggested that humans evolved the propensity to overcome this free rider problem, it is unclear how this module might manifest at the group level. In this study, we investigate the degree to which native and non-native speakers of accents – which are candidates for tags of group membership – spoken in the UK and Ireland can detect mimicry. We find that people are, overall, better than chance at detecting mimicry, and secondly we find substantial inter-group heterogeneity, suggesting that cultural evolutionary processes drive the manifestations of cheater detection. We discuss alternative explanations and suggest avenues of further inquiry.

## Graphical abstract

**Figure:**
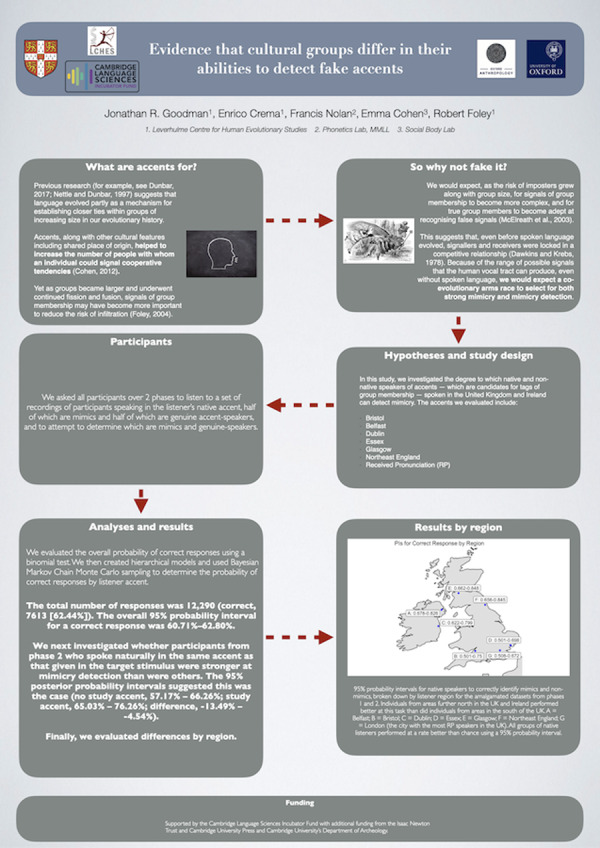

**Social media summary:** New research shows that people are good at telling when someone fakes their accent, and this ability varies by region

How good are humans at identifying cheaters? Previous research has provided mixed findings: people do not detect lying at a consistently higher-than-chance rate across controlled trials (Bond and DePaulo, 2008). Recognising and thwarting free riders, however, is considered in the human evolutionary sciences to have been pivotal in the development of large-scale societies (Tooby and Cosmides, 1992).

One solution to this issue is the evolution of tags, which may allow cooperators – or more specifically, members of cultural groups with high rates of parochial cooperation – to detect one another. McElreath et al. (2003) suggest that clothing styles, tattoos or speech patterns may be tags through which cooperative behaviours are directed. In humans, tags are likely to be inherited socially and signal social identity (see Barth, 1969; Brewer, 1991; Axelrod et al., 2004; see also Smaldino, 2019). This logic helps to explain the evolution of covert signals (‘secret handshakes’; Robson, 1990, see also Smaldino et al., 2018), or signals that represent social identity or group membership, that – ostensibly – only target members of the population recognise and issue (see Adami & Hintze, 2013 and Wiseman & Yilankaya, 2001; see also Pietraszewski, 2022 for a recent and detailed discussion of a computational definition of groups).

Yet if tags are not inflexible phenotypes fixed genetically, cheating represents a significant problem for their implications for human cooperation (McElreath et al., 2003). If, for example, individuals in a cultural group direct altruistic behaviours towards people bearing a group-related tag, such as a tattoo, it is likely that non-group members will mark themselves with the tattoo to free-ride on group benefits, without themselves directing altruism towards true group members (Dawkins et al., 1979; Ruxton et al., 2018).

Evolutionary psychologists have suggested, as a potential solution to the free-rider problem, that a general cheater detection module may thwart free riding across human cooperative networks. Insofar as individuals can recognise and eliminate cheaters from groups, the inclusive benefits of tag-based cooperation are likely to remain unadulterated (Cosmides & Tooby, 1992). Research into false laughter (Bryant & Aktipis, 2014) and fake cooperative intent (Verplaetse et al., 2007) suggests, in line with this general hypothesis, that humans are better than chance at recognising social deception, although the rate of detection is generally about 60–70%. Some deceivers, by implication, evade detection – although the interplay between cooperation-related deception and detection is not well established.

With tags of social origins or identity, furthermore, we should not expect that individuals across social groups are equally good at mimicry detection. Any intergroup variance may therefore suggest culturally specific qualities to thwart free riding. The process of ethnification (where a group becomes phenotypically distinct from others; see Barth, 1969; Boyd & Richerson, 1987; Gil-White, 2001; Bell & Paegle, 2021; Tucker et al., 2021) may play a role in explaining inter-group variance in the detection of mimicry of identity-specific signals. It is possible that varying cultural contexts determine the local emphasis placed on the similarity of accent of speaker and receiver (see Cohen, 2012; Cohen & Haun, 2013), depending on local cultural diversity, cultural boundaries and any local manifestations of parochial altruism relying on social identity signals.

Cohen (2012; see also Cohen & Haun, 2013; Padilla-Iglesias et al., 2020; Cohen et al., 2021) has suggested that accents of languages may be candidates of such tags. Furthermore, accents, unlike laughter, are specific to cultural groups and vary significantly even within subcultures (Nettle, 1999). The causes of cultural diversity, of which linguistic and accent diversity is a part, probably vary by ecological and sociological conditions (Collard & Foley, 2002; Foley, 2004; Foley & Lahr, 2011), but stochastic drift (Nettle, 2012), prestige bias (Henrich & McElreath, 2003) and functional and social (Nettle, 1999, 2012) selection are likely candidates. These processes introduce change in accents both at the group and individual levels. Individuals, for example, are known to change their accents over their lifespan (Evans & Iverson, 2007), and the ability to effectively perceive regional dialects is known to improve through adolescence to adulthood (McCullough et al., 2019).

The diversity and complexity of accents, as well as their importance as markers in modern Western societies (Kinzler, 2021), suggests their recognition may function, at least partly, as a mechanism for directing parochial altruism. Previous work (see Cohen, 2012) suggests that accents may help individuals to do so: they are salient, properties of an individual, readily discriminable, dynamic and universal across cultures, among other qualities.

More broadly, while accents of languages may initially have evolved through drift after group dispersal (see Nettle, 1999), they may directly or indirectly have made possible social categorization (Pietraszewski & Schwartz, 2014a, b). Insofar as individuals present a social identity, indicating social origins, through accent-related signals, listeners might use these signals to inform partner choice, or bias judgments about speakers more broadly, with the consequence that listeners may emphasise or de-emphasise markers of their own cultural group, including accent, in response (Barth, 1969; see also Smaldino, 2019).

These hypotheses are supported by research in sociolinguistics over the 75 years. Labov's (1963) classic study showed, for example, that the strength of a local accent speaker's linguistic markers can correlate with their own views of their social identity with reference to outsiders. This was shown in Martha's Vineyard, an island off the northeast US coast, and was reproduced in several studies (see, for example, Chambers, 1995). A study by Bourhis and Giles (1977) showed that Welsh speakers emphasised their local accents when interrogated by an aggressive English interlocutor in an experimental setting. More recent studies have repeatedly shown that an individual's accent can drive a listener's perceptions of the speaker's personal or physical characteristics, and even their trustworthiness, regardless of whether the listener is a child or adult (Kinzler et al., 2011; see Giles & Billings, 2004 for a review).

For a feature to qualify as a tag in the evolutionary sense it must, however, be difficult to fake, or honest, to use the terminology from signalling theory (Zahavi, 1975), which prevents free riding by cheaters – although few data exist to date that have helped to verify this premise. Similarly, previous work has not explored how group-level differences in detection of accent mimicry manifest, or the reasons for those manifestations, if they exist.

In the present study, we explored this set of issues over two experiments, which aimed to verify the overall rate of accent mimicry-detection in a large cohort of participants, and to determine inter-group variation in such detection. We have two hypotheses, which we explore and discuss further throughout this paper:

H1: Individuals across groups are likely to be better than chance at mimicry detection;

H2: Individuals with social identities matching the target mimicry identity are likely to be better than others at mimicry detection.

We tested these hypotheses by recruiting more than 900 participants from seven areas across the UK and Ireland to mimic, and attempt to detect mimicry of, the accents spoken in their own home areas. We then recruited a larger group of participants online, which both improved the sample sizes from these seven areas and served to create a control group for comparison.

Our results are in line with expectations with our hypotheses; notably, while participants across groups were better than chance at detecting accent mimicry, participants who spoke naturally in a stimulus's target accent often performed better than others at the accent recognition test.

## Methods

**Figure 1.**
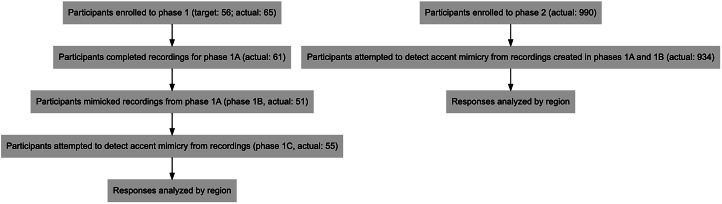
Flowchart of phases 1 and 2 as described in the Methods.

We ran this experiment in two separate phases (Figure 1). In the first, we created a series of sentences to be recorded by speakers of seven accents of English, which varied in the ranges of their geographic usage. We used the following accents: northeast England, Belfast, Dublin, Bristol, Glasgow, Essex and received pronunciation (RP), commonly understood as standard British English. We asked participants in phase 1 to read the sentences outlined in the Supplement §1, which we designed to elicit phonetic variables distinguishing between our accents of interest, using Wells (1982).

We received ethical clearance from the University of Cambridge (Graduate Education Committee, August 2021); all data were, per protocol, de-identified and stored on a Cambridge University cloud system, and were password protected. All de-identified data are available on Github (https://github.com/jonathanrgoodman/accents-2).

### Phase 1 design and recruitment

We divided phase 1 into three parts. In phase 1A, speakers (*n* = 8; four male and four female for each accent of interest, for an intended phase 1 accrual of 56 participants) read a set of sentences recorded using the Qualtrics survey platform (https://www.qualtrics.com) with a plug-in from Phonic.Ai (https://www.phonic.ai) to allow for user recording. Participants used their own home hardware, which could have been a mobile device or desktop computer. We then chose the six recordings (three for each sex per accent, comprising 42 total recordings) that we believed best represented the accents of interest for use in phase 1B (see the Supplement §1). We chose only two recordings for females from Bristol because of low accrual. We also included four females and two males from Glasgow because of accrual problems.

In phase 1B, the same participants recruited to 1A were asked to mimic 12 of the selected sentences in the other of the six accents in which they did not speak naturally, chosen randomly. For example, a participant from northeast England mimicked two random recordings each from speakers from Belfast, Dublin, Bristol, Glasgow and Essex and RP. Females mimicked females; males mimicked males.

Each participant was given two tries per sentence in case of error or poor recording quality. We then chose six mimicry attempt recordings for each accent (three for males, three for females) that we believed best approximated the accents in question. This was based on judgments made by the study authors regarding successful reproduction of the phonetic variables of interest at the sentence level (see the Supplement §1 for details).

Finally, in phase 1C, the same participants again were asked to listen to mimicked recordings from other participants of their own accents of both genders and to determine (a) whether the mimicry recordings were fake, (b) their confidence in this response, measured on a confidence scale from 1–3 and (c) whether the accent appeared to be ‘strong’ or ‘weak’.

All participants were asked to determine whether the speaker was an accent-mimic for each of 12 recordings (six mimics and six genuine speakers, presented in random order). We coded sentence-level scores as ‘1’ or ‘0’ for whether mimics were correctly identified and where genuine accent-speakers were correctly identified, and for false-positives and false-negatives, respectively. The maximum score on this task was 12. Listeners were asked to listen to each stimulus twice.

Because of the study design, it was possible for a participant to hear their own voice during the exercise. For this reason, in phase 1C, we had an additional possible check box indicating ‘I think this is my own voice’. We removed answers where this box was checked; we also checked responses to ensure that no participant heard their own voice and did not check the box. Also, as we chose those recordings that best represented the accent in question, males and females did not hear the same recordings on task 1C. While it was possible for participants to hear the same stimulus in tasks 1B and 1C, the phases took place over one month apart, and given the recordings were on average 2 seconds long, we do not believe this creates any noise in our data.

Finally, we asked participants to provide basic demographic information. Further information about sentence selection and the Qualtrics system can be found in the online supplementary material on Github (https://github.com/jonathanrgoodman/accents-2). All recordings from phases 1A–1C were edited using Apple software (iMovie) to eliminate background noise and normalize volumes.

We attempted to recruit individuals to phase 1 through university listservs in the UK and Ireland, and through traditional media, including local newspapers and radio. Participants were able to participate digitally only; each participant completed a written or digital consent form and had a chance to receive one of seven available Amazon gift cards. All Qualtrics surveys in phase 1 were e-mail-invite only.

### Phase 2 design and recruitment

In phase 2, we aimed to recruit a larger group of participants from the UK and Ireland, regardless of which accent they spoke naturally (our target accrual was 1000 individuals). Participants could participate only on an open Qualtrics survey that prevented multiple accesses from the same IP address.

Each participant was asked for basic demographic data, including whether they spoke in any of our accents of interest (northeast England, Belfast, Dublin, Bristol, Glasgow, Essex and RP) naturally. If they selected one of these accents, they received the identical task to participants in phase 1C.

If they selected that they did not speak in any of these accents, they received a random sample of 14 (one genuine, one fake for seven accents) recordings, presented in a random order. As in phase 1C, we asked each participant to answer ‘yes’ or ‘no’ about whether each recording was a mimic, and specified the accent being attempted. Scores were recorded in an identical manner to phase 1C, except that the maximum possible score was 14.

We recruited participants through university listservs as well as through Twitter/X (https://www.twitter.com) using a promoted tweet from the corresponding author's Twitter/X account. All participants completed a digital consent form prior to participation. There was no compensation for this task.

### Analysis

We initially analysed phases 1 and 2 separately. For each phase, we calculated the Jeffreys interval of the overall probability of a correct response. We then fitted Bayesian hierarchical models, using participant and stimulus as random-level effects, to determine whether individuals were, by region, better than chance at detecting mimics.

Next, we amalgamated data from both phases and fitted a further Bayesian hierarchical model to determine whether:

- individuals were better than chance at detecting mimics overall (H1);
- whether an individual who spoke naturally in a target accent was better at mimicry detection than was an individual not from that region (H2); and
- whether the likelihood of a correct response differed by accent-region.

We completed these statistical analyses and visualisations using the R statistical computing language (R Core Team 2021) using the *brms* (Bürkner et al., 2022), *tidyr* (Wickham et al., 2022), *ggplot2* (Wickham, 2016) and *ggridges* (Wilke, 2021) packages. R scripts are available on the study's Github page. All models included slopes for random effects.

## Results

### Phase 1

#### Participants

While our intended accrual was eight participants per seven accents (56 total), 71 people wrote to express interest in participating; 65 completed the consent form and 61 completed phase 1A; 51 and 55 completed phases 1B and 1C, respectively. As we faced low accrual initially, and were concerned about losing participants to follow-up, we did not limit enrolment to eight individuals per accent. Participants confirmed that they spoke in the relevant accent of interest; we separately confirmed this when listening to phase 1A recordings. Before moving to phase 1B, we ensured we had at least six usable recordings from unique participants (three for each sex) for each accent.

Of participants who completed phase 1C, 33 and 22 participants identified as female and male sex, respectively; see Table 1 for further demographic data. Participants who did not complete any of the phases were removed from the database; in four cases, participants did not complete phase 1B but did complete phase 1C, and their data were included in the analysis.

**Table 1.** Demographic data for participants included in phase 1C (F = female; M = male)

Age (median [range]; years)	41 (18–83)
Sex (female; *N* (%))	33 (60%)
Sex (male; *N* (%))	22 (40%)
Accents (*N* (*N* female, *N* male))	
Belfast	5 (3 F, 2 M)
Bristol	6 (3 F, 3 M)
Dublin	9 (5 F, 4 M)
Essex	11 (7 F, 4 M)
Glasgow	5 (4 F, 1 M)
Northeast	7 (5 F, 2 M)
Received pronunciation	12 (7 F, 5 M)

#### Analysis

The total number of responses to the mimicry detection task in phase 1C was 618, of which 424 (68.61%) answers were correct; this corresponded to an overall 95% Jeffrey's probability interval (PI) of 63.24–72.20%, indicating a better-than-chance ability to detect mimics in the overall phase 1 cohort.

**Figure 2.**
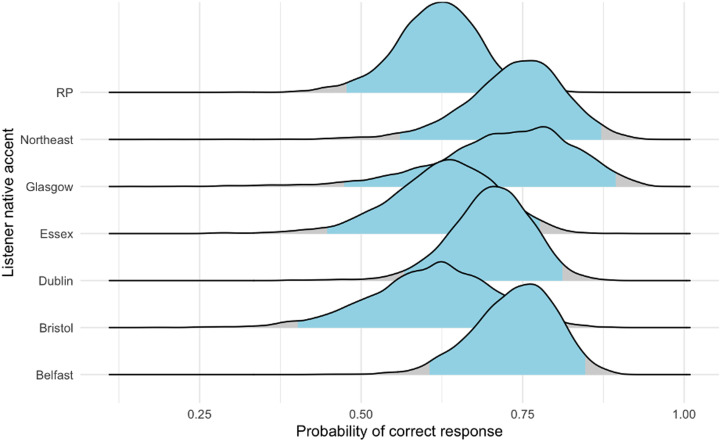
Probability intervals for correctly identifying mimics and non-mimics by listener region; individuals heard only the target accent with which they identified speakers (see Methods).

**Figure 3.**
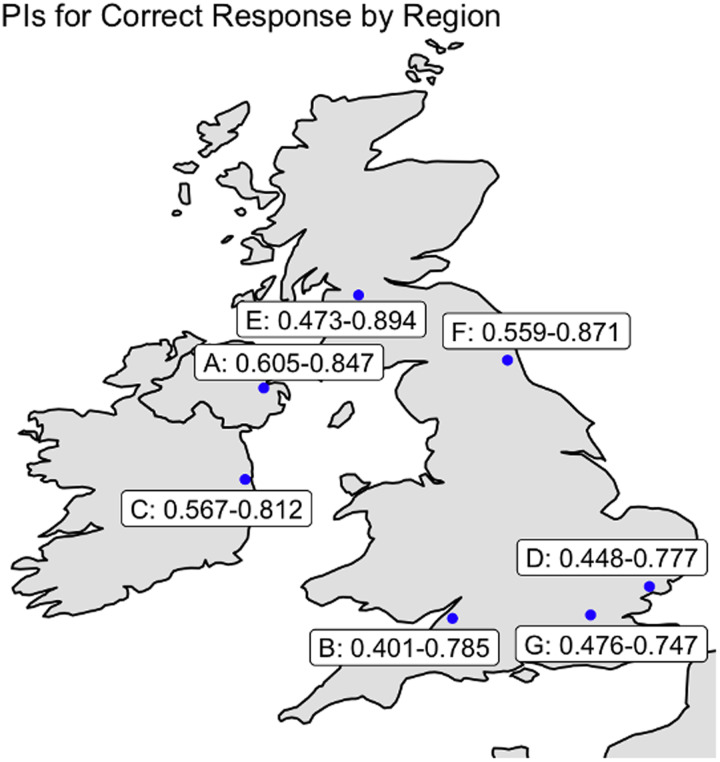
Probability intervals for correctly identifying mimics and non-mimics by listener region from phase 1; individuals heard only the target accent with which they identified speakers (see Methods). Individuals from areas further north in the UK and Ireland performed better at task phase 1C (identifying mimics and non-mimics of their home target accents) than did individuals from areas in the south of the UK. A, Belfast; B, Bristol; C, Dublin; D, Essex; E, Glasgow; F, northeast England; G, London (the city with the most received pronunciation speakers in the UK).

To determine the probability of correctly detecting mimics by participant accent–group, we fitted a Bayesian hierarchical model using Markov Chain Monte Carlo sampling. The model's formula, priors, and other details are provided in Table 2 (Model 1); further data are available in the online supplementary material. Individuals speaking in accents from cities located further north in the UK and Ireland had higher probabilities of a correct response than did individuals from cities in the south (Figures 2 and 3).

**Table 2.** Summary of Markov Chain Monte Carlo model (phase 1)

Model	Formula	Dependent variable	Fixed effects	Random-level effects	Prior	Bulk effective sample size	Rhat	Posterior predictive check	95% probability interval of estimate	95% probability interval of estimated probability of success
1	Correct~(Accent-1)+ (1|Participant)+ (1+Accent|Question)	Correct detection of mimic or genuine speaker	Participant accent	Participant, stimulus	Normal(0,1)	All > 1000	All = 1.00	See online supplementary material	See online supplementary material	See Figures 2 and 3

### Phase 2

#### Participants

Of 1709 participants who visited the Qualtrics-hosted study, 990 completed the online consent form; the basic demographic data are given in Table 3. All participants verified they were from the UK or Ireland. Forty-nine participants did not respond to the question regarding their natural accent and seven did not respond to any accent-mimicry question; this left 934 participants for the overall phase 2 analysis.

**Table 3.** Participant demographics for phase 2

Age (median (range); years)	48 (17–85)
Sex (female; *N* (%))	471 (47.58%)
Sex (male; N (%))	452 (45.66%)
Sex (Other/non-binary; N (%))	14 (1.41%)
Sex (unknown; *N* (%))	53 (5.54%)
*Accents (N)*	
Belfast	28
Bristol	7
Dublin	10
Essex	22
Glasgow	44
Northeast	33
Received pronunciation	182
None of the above	615
Unknown	49

#### Analysis

The total number of responses to the mimicry detection task in phase 2 was 11,672, of which 7189 (61.59%) answers were correct; this corresponded to an overall 95% probability interval of correctly identifying a mimic or non-mimic of 60.32–62.47%, a finding comparable with that seen with the smaller sample size of phase 1.

**Figure 4.**
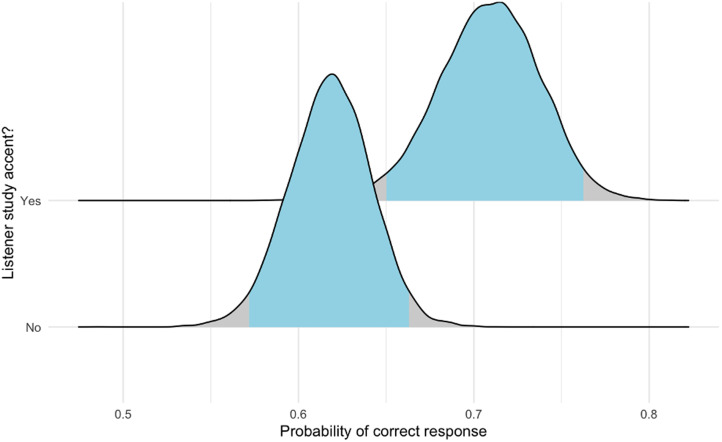
Probability of correct response in phase 2 by whether participants who spoke naturally in one of our seven study accents (Belfast, Bristol, Dublin, Essex, Glasgow, northeast England and received pronunciation) were, overall, better at the task than were participants who did not speak naturally in one of these accents. The posterior probability intervals suggested that this was the case (no study accent, 57.17–66.26%; study accent, 65.03–76.26%; difference, −13.49 to −4.54%).

As with the initial dataset from phase 1C, we fitted a Bayesian hierarchical model using Markov Chain Monte Carlo sampling to the phase 2 data. Here, we investigated whether participants from phase 2 who spoke naturally in the same accent as that given in the target stimulus were stronger at mimicry detection than were others. The posterior probability intervals suggested that this was the case (no study accent, 57.17–66.26%; study accent, 65.03–76.26%; difference, −13.49 to −4.54%; Figure 4). The model's formula, priors, PIs and other details are provided in Table 4 (Model 2).

**Table 4.** Model from the phase 2 dataset (see main text)

Model	Formula	Dependent variable	Fixed effects	Random-level effects	Prior	Bulk effective sample size	R-hat statistic	Posterior predictive check	95% probability interval of estimate	95% probability interval of estimated probability of success
2	Correct~(Study.accent-1)+ (1|Participant)+ (1+Accent|Question)	Correct detection of mimic or genuine speaker	Binary variable of whether listener spoke naturally in a study accent of interest (see main text)	Participant, stimulus	Normal(0,1)	All > 1000	All < 1.02	See online supplementary material	See online supplementary material	See main text and Figure 4

### Phases 1C and 2 (combined)

Next, we amalgamated responses from phases 1C and 2. Given the experimental setting was identical between the two phases, we combined the data into a single data frame rather than conducting a meta-analysis of the two phases.

**Figure 5.**
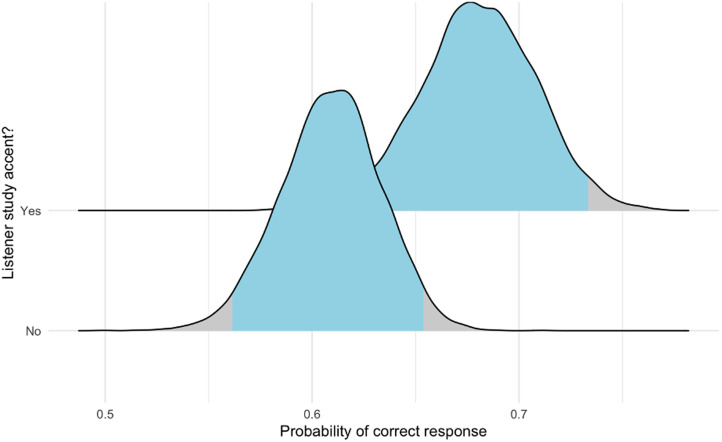
Probability of correct response (using amalgamated data from phases 1C and 2) by whether participants who spoke naturally in one of our seven study accents (Belfast, Bristol, Dublin, Essex, Glasgow, northeast England and received pronunciation) were, overall, better at the task than were participants who did not speak naturally in one of these accents. The posterior probability intervals suggested that this was the case (no study accent, 56.11–65.36%; study accent, 62.46–73.34%; difference, −12.13 to −2.17%).

**Figure 6.**
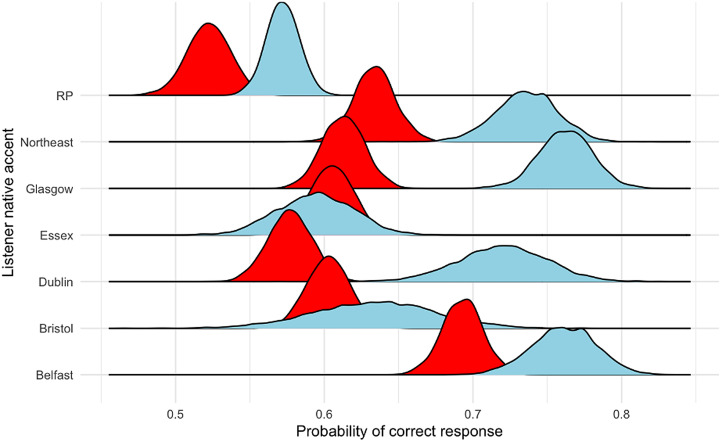
Probability of correct response by region and by whether listeners spoke naturally in the accent of interest. Red, participant did not speak naturally in the relevant accent as given on the *y*-axis; blue, participant spoke naturally in the relevant accent.

**Figure 7.**
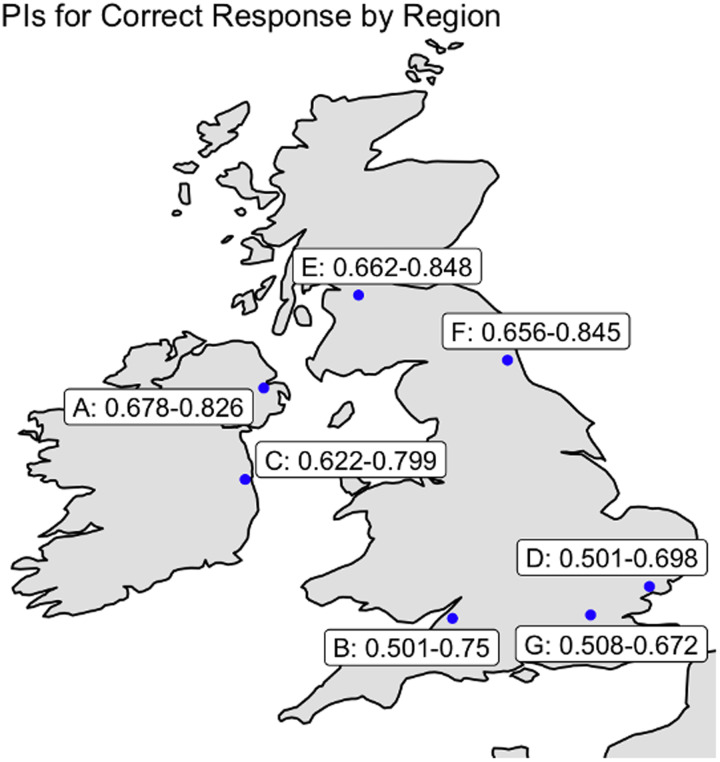
The 95% probability intervals for native speakers to correctly identify mimics and non-mimics, broken down by listener region for the amalgamated datasets from phases 1 and 2. Individuals from areas further north in the UK and Ireland performed better at this task than did individuals from areas in the south of the UK. A, Belfast; B, Bristol; C, Dublin; D, Essex; E, Glasgow; F, northeast England; G, London (the city with the most received pronunciation speakers in the UK). All groups of native listeners performed at a rate better than chance using a 95% probability interval.

The total number of responses was 12,290 (correct, 7613 (62.44%)). The overall 95% probability interval for a correct response was 60.71–62.80%. We fitted models to investigate the overall difference in probability of a correct answer by whether the listener spoke naturally in a study accent (Model 3) and then investigated these results by region (Model 3.1). The findings from Model 3 confirmed those of Model 2 (Figure 5); Model 3.1 suggested variation between our areas of interest. See the probability interval details in Figures 6 and 7 and in Table 5; the models’ formulae, priors and other details are provided in Table 6 and in the online supplementary material. Three accents of interest – Dublin, Glasgow and northeast England – showed a non-zero difference between natural speakers and non-natural speakers in the likelihood of detecting mimics and non-mimics. Importantly, furthermore, all native listener groups performed better than chance using a 95% probability interval; this was not true of all non-native listener groups.

**Table 5.** Probability intervals (PIs) for a correct response by whether individuals spoke in a study accent, broken down by accent group (the left-most column indicates the difference). All groups of native listeners performed at a rate better than chance using a 95% probability interval

Accent	Listener does not speak naturally in stimulus accent	Listener speaks naturally in stimulus accent	Difference
Belfast	59.13–75.91%	67.85–82.63%	−16.45–0.69%
Bristol	47.90–70.47%	50.12–74.96%	−18.14–11.05%
Dublin	45.81–67.69%	62.22–79.89%	−26.49 to −2.77%
Essex	51.51–72.63%	50.06–69.81%	−9.28–13.24%
Glasgow	48.00–70.80%	66.23–84.78%	−28.68 to −4.08%
Northeast England	52.36–73.02%	65.65–84.47%	−22.96 to −2.26%
Received pronunciation	42.27–64.09%	50.76–67.19%	−16.39–4.51%

**Table 6.** Models from amalgamated dataset (see main text)

Model	Formula	Dependent variable	Fixed effects	Random- level effects	Prior	Bulk effective sample size	Rhat	Posterior predictive check	95% probability interval of estimate	95% probability interval of estimated probability of success
3	Correct~ (Study.accent-1)+ (1|Participant)+ (1+Accent|Question)	Correct detection of mimic or genuine speaker	Binary variable of whether listener spoke naturally in a study accent of interest (see main text)	Participant, stimulus	Normal(0,1)	All > 500	All < 1.02	See online supplementary material	See online supplementary material	See Figure 5
3.1	Correct~ (Study.accent-1): (Sentence.accent-1)+ (1|Participant)+ (1+Accent|Question)	Correct detection of mimic or genuine speaker	Interaction between fixed effect from Model 3 and target study accent	Participant, stimulus	Normal(0,1)	All > 500	All < 1.02	See online supplementary material	See online supplementary material	See Figure 6 and Table 5

## Discussion

In the present set of studies, we found that: (a) participants from phases 1 and 2 were, across groups, better than chance at detecting accent mimicry, supporting H1; (b) participants who spoke natively in the mimicry target accent were better than non-natives at detecting mimicry, supporting H2; and (c) that there was substantial heterogeneity in detecting mimics among groups. Overall, while mimicry detection was greater than chance across groups, we saw high variance based on participant qualities, including the native accent group and whether listeners spoke natively in the target accent of mimicry. Native listeners were consistently better than chance at detecting mimics across groups; this was not true among non-native listeners.

Unlike with previous research into free rider detection, which has looked into laughter (Bryant & Aktipis, 2014), lying (Fonseca & Peters, 2021) and cooperative intent (Verplaetse et al., 2007), and which moreover found comparable overall rates of detection (~60–70%), we evaluated detection quality differences by group, and found substantial intergroup variance. This suggests that cheating detection is context dependent, and may be the result of ethnification processes and cultural transmission.

The view that accents evolved as signals to better allow phenotypic matching – whether for kinship recognition (see Cohen, 2012), to determine likelihood of cooperative intent (Verplaetse et al., 2007) or to determine social identity (Smaldino, 2019; Pietraszewski & Schwartz, 2014a, b) – cannot, we suggest, alone explain the variation in intergroup mimicry detection we find here. It is possible, for example, that in our ancient history, accents evolved as signals indicating kinship, much as other forms of phenotypic matching may serve as signals of shared ancestry (Lieberman et al., 2007). It is likely that while stochastic drift played a role in changes in linguistic traits associated with accents as groups lived separately over time, accents underwent ritualization (see Tinbergen, 1952) as individuals who used these cues for phenotypic matching gained inclusive fitness benefits through reciprocal cooperative preferences (Humphrey, 1997). Given previous work suggesting that groups reproducing in isolation are likely to have a higher than total population average genetic relationship (Lehmann et al., 2007), the inclusive fitness benefits of phenotypic matching through accent detection are likely to be non-zero.

As with any evolutionary model where social gains are possible through signalling, free riding presents a problem that only effective detection may solve (Goodman & Ewald, 2021; for a model, see Goodman, 2023). This general kinship detection model, while providing a plausible ultimate explanation for the view that accents are signals, does not make any prediction about intergroup or intercultural variation in cheater detection. We suggest that sociocultural factors explain this set of findings, and that detection may allow for frequency-dependent free-riding (see Sperber & Baumard, 2012), which will vary by local cultural norms and boundaries.

Our finding that there was heterogeneity in detection rates among groups may provide evidence in favour of this view. Timur Kuran (1998) gave a model suggesting that, in terms of ethnic norms, an individual's utility function is determined largely by reputational concerns. Norms that are not associated with ethnicity (for example, wearing a homespun, cheap hat) can undergo ethnification insofar as the norm becomes associated with an ethnic group – and further, members of the ethnic group, because of sociocultural reasons, begin to place emphasis on practising ethnicity-specific norms. Kuran notes, for example, that towards the end of British rule in India, homespun hats worn by Hindus became known as ‘Gandhi caps’; Muslims, in contrast, began to place cultural emphasis on wearing fur hats. While hat choices, prior to the ethnoreligious divides that became pervasive in India during this period, were once indicative only of what a person could afford, the tensions between India's two largest ethnoreligious groups created individual-level pressures to practice these norms as ethnic signals. Individuals who did not change their practices to identify with the norms of their ethnoreligious group suffered potential reputational damage – creating a cascade effect that more closely linked hat-wearing and group-level tensions and biases.

We suggest that an analogous phenomenon may explain the intergroup variance in mimicry detection noted in our models. Sociocultural events, such as between-group competition, may cause cultural selection processes to speed up (Henrich & Muthukrishna, 2021), forcing a greater focus on detecting out-group members (Goodman et al., 2023). Even if functional selection and stochastic drift (Nettle, 1999) lead to greater differences between groups in linguistic traits, increasing animosity and social boundaries may not only speed up such change through social selection, but also place greater emphasis on both manifesting and detecting culturally relevant norms at the individual level (see Labov, 1963; Giles, 1977; and Chambers, 1995 for examples of changes in accent-manifestation because of between-group animosity). Implicit to Kuran's model is recognition by individuals of Gandhi caps as ethnoreligious signals informing as to personal bias; accents, similarly, may become of increasing social importance insofar as the identities they signal have consequences for speaker and listener. Similar contextualization is critical in multi-linguistic settings where accents are less important signals of social identity (Cohen & Haun, 2013). The ethnification of accents may be a consequence of between-group forces imposing cultural selection on both linguistic traits and their recognition – a cultural form of the ritualization of cues described by Tinbergen (1952).

The accents of speakers from Belfast, Glasgow, Dublin and northeast England have culturally evolved over the past several centuries, during which time there have been multiple cases of between-group cultural tension, especially with the cultural group making up southeast England, particularly London. The ethnification processes described by Kuran, and which may be described in the language of cultural evolutionary pressures, probably caused individuals from areas in Ireland and the northern regions of the UK to place emphasis on their accents as signals of social identity. Greater social cohesion among accent speakers may have increased the risks posed by free riders from other groups, necessitating improved accent recognition and mimicry detection – characteristics probably not needed by individuals without strong cultural group boundaries, such as those living in London. (We give a rudimentary analysis evaluating whether ethnic diversity in a region predicts success in our experimental task in Supplement Section 2. Our findings are inconclusive but warrant further study.)

This narrative both predicts better mimicry detection among speakers from places with high-between group tension, such as Belfast, Glasgow and Dublin, and explains why an area like Essex may also have relatively poor mimicry detection. Specifically, speakers of the Essex accent moved to this area over the past 25 years from London (see Watt et al., 2014) – a strong contrast with speakers living in Belfast, Glasgow and Dublin, whose accents evolved over centuries of cultural tension and violence.

Together, these findings lend preliminary support to a model that increases accent recognition and mimicry detection pressures in accordance with cultural evolutionary processes, specifically between-group tension (see Boyd & Richerson, 1998). Rather than assume interindividual variance in cheater detection with a mean rate of 66%, we should view recognition and mimicry detection as functions of sociocultural processes that wax and wane in pressure in accordance with ethnification. A formal model accounting for similar terms to those of Kuran (1998) would explain not only the intergroup variance we find here, but also the differences in the culturally significant accent-signals that make recognition and mimicry detection more likely. Cultural evolutionary processes, based on intergroup relationships, will in our view, select for both linguistic traits likely to differentiate ethnic or cultural groups and cultural learning processes allowing better recognition of signal mimicry.

Finally, we suggest that this set of findings lends support to Nolan's (2012) view that native accent-speakers are likely to be better than non-natives at recognizing natural accents and determining when a speaker is faking a relevant target accent. Hoskin and Foulkes (forthcoming; personal communication) showed, relatedly, that native Syrian individuals and native Syrian linguists are highly effective at determining speaker authenticity in linguistic tests for asylum (recognition rates ~90–100%). Both non-Syrian native speaker non-linguists and non-Syrian linguists, in contrast, had correct rejection rates of ~85–88%. Our results, which are based on shorter sentences than are linguistic tests such as the Language Analysis for the Determination of Origin, which is used in the UK for asylum, suggest, following Nolan (2012), that native accent-speakers are likely to be stronger at mimicry detection than are others. Coupled with Hoskin and Foulkes's findings, it follows that native speakers ought to be used in asylum tests – a suggestion that should be explored in future research into mimicry of linguistic signals.

Our study has several limitations that may be addressed in future experiments. For example, we conducted our studies only using participants from the UK and Ireland, and specifically using only seven accents from these countries. Moreover, some features of the accents we chose may have affected our findings, such as how familiar listeners and speakers were with some of the chosen accents, such as RP. Similarly, heterogeneity within accent groups, such as northeast England, may have affected our findings. Finally, our sub-group sample sizes, particularly for some native-speaker groups like Bristol, prevents our testing for interactions between whether a listener is a native speaker and region.

While these limitations prevent broad generalization of the model we are advocating here, future studies may explore these aspects further, such as whether our findings apply to other cultural groups in other regions who speak different languages, and whether our findings hold in a follow-up experiment with a larger sample size. We believe, however, that research in the US (Tate, 1979) and into asylum tests of individuals from Syria (Hoskin and Foulkes, forthcoming) – the latter of which explored a fuller diagnostic test than the short recognition test performed here – points to broad support for our general findings.

We also suggest that future experiments account for how individual biases evolve based on signals of social identity in response to changing group relationships. While previous research has established that such signals drive preferential treatment (Kinzler, 2021), and even the perception of veracity (Lev-Ari & Keysar, 2010), it is unknown, following the model we espouse here, whether evolving relationships between groups, and the consequent ethnification of group-level signals, directly affect interpersonal treatment. We suggest that economic games are used to explore this further.

## Supporting information

Goodman et al. supplementary material Goodman et al. supplementary material

## Data Availability

All information about the study's design and protocols, as well as the data and analyses associated with this research are available at https://github.com/jonathanrgoodman/accents-2.

## Supplementary material

To view supplementary material for this article, please visit https://doi.org/10.1017/ehs.2024.36

## References

[ref1] Adami, C., & Hintze, A. (2013). Evolutionary instability of zero-determinant strategies demonstrates that winning is not everything. Nature Communications, 4(1), article 1. 10.1038/ncomms3193 PMC3741637 23903782

[ref2] Axelrod, R., Hammond, R. A., & Grafen, A. (2004). Altruism via kin-selection strategies that rely on arbitrary tags with which they coevolve. Evolution; International Journal of Organic Evolution, 58(8), 1833–1838. 10.1111/j.0014-3820.2004.tb00465.x 15446434

[ref3] Barth, F. (1969). Ethnic groups and boundaries: The social organization of culture difference. Little, Brown.

[ref4] Bell, A. V., & Paegle, A. (2021). Ethnic markers and how to find them. Human Nature, 32(2), 470–481. 10.1007/s12110-021-09401-z 34105061 PMC8186961

[ref5] Bond, C. F., & Depaulo, B. M. (2008). Individual differences in judging deception: accuracy and bias. Psychological bulletin, 134(4), 477–492. 10.1037/0033-2909.134.4.477 18605814

[ref6] Bourhis, R. Y., & Giles, H. (1977). The language of intergroup distinctiveness. In H. Giles (Ed.), Language, ethnicity and intergroup relations (pp. 119–135). Academic Press.

[ref7] Boyd, R., & Richerson, P. J. (1987). The evolution of ethnic markers. Cultural Anthropology, 2(1), 65–79.

[ref8] Boyd, R., & Richerson, P. J. (1988). Culture and the evolutionary process (2nd ed.). University of Chicago Press.

[ref9] Brewer, M. B. (1991). The social self: On being the same and different at the same time. Personality and Social Psychology Bulletin, 17(5), 475–482. 10.1177/0146167291175001

[ref10] Bryant, G. A., & Aktipis, C. A. (2014). The animal nature of spontaneous human laughter. Evolution and Human Behavior, 35(4), 327–335. 10.1016/j.evolhumbehav.2014.03.003

[ref11] Bürkner, P.-C., Gabry, J., Weber, S., Johnson, A., Modrak, M., Badr, H. S., …, Mills, S. C. (2022). *brms: Bayesian Regression Models using ‘Stan’* (2.18.0). https://CRAN.R-project.org/package=brms

[ref12] Chambers, J. K. (1995). Sociolinguistic theory: Linguistic variation and its social significance. Wiley.

[ref13] Cohen, E. (2012). The evolution of tag-based cooperation in humans: The case for accent. Current Anthropology, 53(5), 588–616. 10.1086/667654

[ref14] Cohen, E., & Haun, D. (2013). The development of tag-based cooperation via a socially acquired trait. Evolution and Human Behavior, 34(3), 230–235. 10.1016/j.evolhumbehav.2013.02.001

[ref15] Cohen, E., van Leeuwen, E. J. C., Barbosa, A., & Haun, D. B. M. (2021). Does accent trump skin color in guiding children's social preferences? Evidence from Brazil's natural lab. Cognitive Development, 60, 101111. 10.1016/j.cogdev.2021.101111

[ref16] Collard, I. F., & Foley, R. A. (2002). Latitudinal patterns and environmental determinants of recent human cultural diversity: Do humans follow biogeographical rules? Evolutionary Ecology Research, 4(3), 371–383.

[ref17] Cosmides, L., & Tooby, J. (1992). Cognitive adaptations for social exchange. In J. H. Barkow, L. Cosmides, & J. Tooby (Eds.), The adapted mind: Evolutionary psychology and the generation of culture (pp. 163–228). Oxford University Press.

[ref18] Dawkins, R., Krebs, J. R., Maynard Smith, J., & Holliday, R. (1979). Arms races between and within species. Proceedings of the Royal Society of London. Series B. Biological Sciences, 205(1161), 489–511. 10.1098/rspb.1979.0081 42057

[ref19] Evans, B. G., & Iverson, P. (2007). Plasticity in vowel perception and production: a study of accent change in young adults. The Journal of the Acoustical Society of America, 121(6), 3814–3826. doi:10.1121/1.2722209 17552729

[ref20] Foley, R. A. (2004). The evolutionary ecology of linguistic diversity in human populations. In M. Jones (Ed.), Traces of ancestry: Studies in honour of Colin Renfrew (pp. 61–71). McDonald Institute for Archaeological Research.

[ref21] Foley, R. A., & Mirazón Lahr, M. (2011). The evolution of the diversity of cultures. Philosophical Transactions of the Royal Society B: Biological Sciences, 366(1567), 1080–1089. 10.1098/rstb.2010.0370 PMC3049104 21357230

[ref22] Fonseca, M. A., & Peters, K. (2021). Is it costly to deceive? People are adept at detecting gossipers’ lies but may not reward honesty. Philosophical Transactions of the Royal Society of London. Series B, Biological Sciences, 376(1838), 20200304. 10.1098/rstb.2020.0304 34601910 PMC8487739

[ref23] Gil-White, F. J. (2001). Are ethnic groups biological ‘species’ to the human brain? Essentialism in our cognition of some social categories. Current Anthropology, 42(4), 515–553. 10.1086/321802

[ref24] Giles, H. (1977). Language, ethnicity and intergroup relations. Academic Press.

[ref25] Giles, H., & Billings, A. C. (2004). Assessing language attitudes: Speaker evaluation studies. In A. Davies, & C. Elder (Eds.), The handbook of applied linguistics (pp. 187–209). Wiley-Blackwell Publishing Ltd.

[ref26] Goodman, J. R. (2023). The problem of opportunity. Biology & Philosophy, 38, 48. 10.1007/s10539-023-09936-8

[ref27] Goodman, J. R., & Ewald, P. W. (2021). The evolution of barriers to exploitation: Sometimes the Red Queen can take a break. Evolutionary Applications, 14(9), 2179–2188. 10.1111/eva.13280 34603491 PMC8477591

[ref28] Goodman J. R., Caines A., & Foley R. A. (2023). Shibboleth: An agent-based model of signalling mimicry. PLoS ONE 18(7): e0289333. 10.1371/journal.pone.0289333 37523380 PMC10389733

[ref29] Henrich, J., & McElreath, R. (2003). The evolution of cultural evolution. Evolutionary Anthropology: Issues, News, and Reviews, 12(3), 123–135. 10.1002/evan.10110

[ref30] Henrich, J., & Muthukrishna, M. (2021). The origins and psychology of human cooperation. Annual Review of Psychology, 72(1), 207–240. 10.1146/annurev-psych-081920-042106 33006924

[ref31] Humphrey, N. K. (1997). Varieties of altruism – And the common ground between them. Social Research, 64(2), 199–209.

[ref32] Kinzler, K. D. (2021). Language as a social cue. Annual Review of Psychology, 72, 241–264. 10.1146/annurev-psych-010418-103034 33400567

[ref33] Kinzler, K. D., Corriveau, K. H., & Harris, P. L. (2011). Children's selective trust in native-accented speakers. Developmental Science, 14(1), 106–111. 10.1111/j.1467-7687.2010.00965.x 21159092

[ref34] Kuran, T. (1998). Ethnic norms and their transformation through reputational cascades. The Journal of Legal Studies, 27(S2), 623–659. 10.1086/468038

[ref35] Labov, W. (1963). The social motivation of a sound change. Word, 19(3), 273–309. 10.1080/00437956.1963.11659799

[ref36] Lehmann, L., Keller, L., West, S., & Roze, D. (2007). Group selection and kin selection: Two concepts but one process. Proceedings of the National Academy of Sciences, 104(16), 6736–6739. 10.1073/pnas.0700662104 PMC1871855 17416674

[ref37] Lev-Ari, S., & Keysar, B. (2010). Why don't we believe non-native speakers? The influence of accent on credibility. Journal of Experimental Social Psychology, 46(6), 1093–1096. 10.1016/j.jesp.2010.05.025

[ref38] Lieberman, D., Tooby, J., & Cosmides, L. (2007). The architecture of human kin detection. Nature, 445(7129), 727–731. 10.1038/nature05510 17301784 PMC3581061

[ref39] McCullough, E. A., Clopper, C. G., & Wagner, L. (2019). Regional dialect perception across the lifespan: Identification and discrimination. Language and Speech, 62(1), 115–136. doi:10.1177/0023830917743277 29188748

[ref40] McElreath, R., Boyd, R., & Richerson, P. J. (2003). Shared norms and the evolution of ethnic markers. Current Anthropology, 44(1), 122–130. 10.1086/345689

[ref41] Nettle, D. (1999). Linguistic diversity. Oxford University Press.

[ref42] Nettle, D. (2012). Social scale and structural complexity in human languages. Philosophical Transactions of the Royal Society B: Biological Sciences, 367(1597), 1829–1836. 10.1098/rstb.2011.0216 PMC3367698 22641821

[ref43] Nolan, F. (2012). Degrees of freedom in speech production: An argument for native speakers in LADO. International Journal of Speech, Language and the Law, 19(2), 263–289. 10.1558/ijsll.v19i2.263

[ref44] Padilla-Iglesias, C., Foley, R. A., & Shneidman, L. A. (2020). Language as a marker of ethnic identity among the Yucatec Maya. Evolutionary Human Sciences, 2. 10.1017/ehs.2020.39 PMC10427450 37588346

[ref45] Pietraszewski, D. (2022). Toward a computational theory of social groups: A finite set of cognitive primitives for representing any and all social groups in the context of conflict. Behavioral and Brain Sciences, 45, e97. 10.1017/S0140525X21000583 33902764

[ref46] Pietraszewski, D., & Schwartz, A. (2014a). Evidence that accent is a dimension of social categorization, not a byproduct of perceptual salience, familiarity, or ease-of-processing. Evolution and Human Behavior, 35(1), 43–50. 10.1016/j.evolhumbehav.2013.09.006

[ref47] Pietraszewski, D., & Schwartz, A. (2014b). Evidence that accent is a dedicated dimension of social categorization, not a byproduct of coalitional categorization. Evolution and Human Behavior, 35(1), 51–57. 10.1016/j.evolhumbehav.2013.09.005

[ref48] R Core Team. (2021). R: A language and environment for statistical ## computing. R Foundation for Statistical Computing, Vienna, Austria.

[ref49] Robson, A. J. (1990). Efficiency in evolutionary games: Darwin, Nash and the secret handshake. Journal of Theoretical Biology, 144(3), 379–396. 10.1016/S0022-5193(05)80082-7 2395377

[ref51] Ruxton, G. D., Sherratt, T. N., & Speed, M. P. (2018). Avoiding attack: The evolutionary ecology of crypsis, warning signals and mimicry. Oxford University Press. 10.1093/acprof:oso/9780198528609.001.0001

[ref52] Smaldino, P. E. (2019). Social identity and cooperation in cultural evolution. Behavioural Processes, 161, 108–116. 10.1016/j.beproc.2017.11.015 29223462

[ref53] Smaldino, P. E., Flamson, T. J., & McElreath, R. (2018). The evolution of covert signaling. Scientific Reports, 8(1), Article 1. 10.1038/s41598-018-22926-1 PMC5861109 29559650

[ref54] Sperber, D., & Baumard, N. (2012). Moral reputation: An evolutionary and cognitive perspective. Mind & Language, 27(5), 495–518. 10.1111/mila.12000

[ref55] Tate, D. A. (1979). Preliminary data on dialect in speech disguise. Current Issues in the Phonetic Sciences: Proceedings of the IPS-77 Congress, Miami Beach, Florida, 17–19 December 1977, pp. 847–850. 10.1075/cilt.9.90tat

[ref56] Tinbergen, N. (1952). A note on the origin and evolution of threat display. Ibis, 94(1), 160–162. 10.1111/j.1474-919X.1952.tb01797.x

[ref57] Tooby, J., & Cosmides, L. (1992). The psychological foundations of culture. In J. H. Barkow, L. Cosmides, & J. Tooby (Eds.), The adapted mind: Evolutionary psychology and the generation of culture (pp. 19–136). Oxford University Press.

[ref58] Tucker, B., Ringen, E. J., Tsiazonera, Tombo, J., Hajasoa, P., Gérard, S., …, Garçon, A. H. (2021). Ethnic markers without ethnic conflict. Human Nature, 32(3), 529–556. 10.1007/s12110-021-09412-w 34546550

[ref59] Verplaetse, J., Vanneste, S., & Braeckman, J. (2007). You can judge a book by its cover: The sequel. A kernel of truth in predictive cheating detection. Evolution and Human Behavior, 28(4), 260–271. 10.1016/j.evolhumbehav.2007.04.006

[ref60] Watt, P., Millington, G., & Huq, R. (2014). East London mobilities: The ‘Cockney diaspora’ and the remaking of the Essex ethnoscape. In P. Watt & P. Smets (Eds.), Mobilities and neighbourhood belonging in cities and suburbs (pp. 121–144). Palgrave Macmillan. 10.1057/9781137003638_7

[ref61] Wells, J. C. (1982). Accents of English (Vol. 2). Cambridge University Press. 10.1017/CBO9780511611759

[ref62] Wickham, H. (2016). ggplot2: Elegant graphics for data analysis. Springer-Verlag. https://ggplot2.tidyverse.org

[ref50] Wickham, H., Girlich, M., & RStudio. (2022). *tidyr: Tidy messy data* (1.2.1). https://CRAN.R-project.org/package=tidyr

[ref63] Wilke, C. O. (2021). *ggridges: Ridgeline plots in ‘ggplot2’* (0.5.3). https://CRAN.R-project.org/package=ggridges

[ref64] Wiseman, T., & Yilankaya, O. (2001). Cooperation, secret handshakes, and imitation in the prisoners’ dilemma. Games and Economic Behavior, 37(1), 216–242. 10.1006/game.2000.0836

[ref65] Zahavi, A. (1975). Mate selection – A selection for a handicap. Journal of Theoretical Biology, 53(1), 205–214. 10.1016/0022-5193(75)90111-3 1195756

