# Supplementary material for: Evidence that cultural groups differ in their abilities to detect fake accents
Source: Evol Hum Sci. 2024 Nov 20;6:e46. doi: 10.1017/ehs.2024.36 (PMC11658943; doi:10.1017/ehs.2024.36)
Supplement: Goodman et al. supplementary material [file S2513843X24000367sup001.docx]

## Supplement

### 1

We designed the below sentences based on Wells (1982) to bring out distinctions between our accents of interest (see main text). The numbered and lettered markings below the phonemes are explains in the *Variables* section.

*Sentences*

They serve such good food in bars

          1,2     3       3,4     4          5,2

Hold up those two cooked tea bags.

6       3    7             4     4          8    5

Who got caught at work first?

   6 4   1     1                 A     A

She kicked the goose hard with her foot.

       1         7       4       6           7         4

His nurse was thinking the cure is a fair price.

6      A,2            7     B      7      F,2         1,2    C

Start their car near the square.

  5,2  7 1,2 5,2   F,2  7        F,2

Marty is a hardy man.

   5 D 8      6 2 D 8   5

He thought a bath would make him happy.

7 E               5,7                 1            6,5   8

Jenny told him to face up to his weight.

            1       1         C     1       1 C I

Kit strutted across the room.

1       3         1          7        1

They removed the palms to build a farm.

7        1     1       7       5                          5,2

The film’s viewership generated a lot of data.

7       G        1     2           1                  1         1 H

*Variables*

1 Quality of vowel

2 ±Non-prevocalic rhotic

3 FOOT-STRUT split

4 FOOT-GOOSE merger

5 Frontness and length of vowel

6 H drop

7 Dental fricative θ, ð vs dental stop t̪, d̪ (f, d, v also known variants)

8 Quality of ‘happY’ vowel (ranging from [ɛ] ~ close [i:])

9 Roundness

A NURSE-NORTH merger

B treatment of final <-ing>

C Variable on [feːs] ~ [wɛɪt] range

D ±Neutralization of /t/ vs /d/ opposition

E ±Long vowel

F Treatment of centering diphthongs

G ±Dark l

H ±Hypercorrect r-insertion

I ±Dropped final /t/ sound

### 2

To explore the possibility that ethnic diversity predicts success in our experimental task, we collected data about ethnodiversity (as measured by the percentage of individuals identifying as White) in the urban areas associated with our accents of interest. We used census data from England and Wales from 2011 (<https://www.nomisweb.co.uk/census/2011/ks201ew>), as well as 2011 data from the Ireland Central Statistics Office (Ireland CSO, 2012) and the Scottish Census Office (Scotland CO, 2012) to measure the percentage of individuals identifying as White ethnicity in the urban areas of Belfast, Bristol, Dublin, Colchester (Essex), Glasgow, and Newcastle (Northeast England), and RP. We fitted a final Bayesian hierarchical model to determine whether the diversity of a given area predicted the likelihood that individuals from that area were strong at mimicry detection.

We excluded RP from this analysis because it is at least arguably non-regional. Furthermore, we excluded Essex because of its unique sociological factors: an influx of cockney-speaking individuals over the past 25 years (the ‘Cockney Diaspora,’ see Watt et al., 2014) suggested an artificial cultural homogeny not present prior to this period. We intended ethnic diversity to be seen as a proxy for cultural diversity in this study; the Cockney Diaspora eliminated the link between ethnicity and cultural diversity in Essex only, which contrasted with the other cities of interest where these demographic factors were absent. The ethnodiversity data we obtained are in **Table S1**; the results from the MCMC models are available on the study’s Github page (<https://github.com/jonathanrgoodman/accents-2>).

While the model was inconclusive owing to the low number of regions, as well as the regions having a minimum percentage of individuals identifying as White of 84%, the results warrant further investigation (see the analyses and figure at <https://htmlpreview.github.io/?https://github.com/jonathanrgoodman/accents-2/blob/main/ethnicity-analyses.nb.html>).

| Accent (City) | Percentage of individuals identifying as White ethnicity |
| --- | --- |
| Belfast | 96.43% |
| Bristol | 84.00% |
| Dublin | 90.40% |
| **Essex (Colchester)*** | **93.30%** |
| Glasgow | 88.40% |
| Northeast England (Newcastle upon Tyne) | 85.30% |
| **RP (London)** | **59.70%** |

Table S1: Diversity data obtained from 2011 census sources (see main text); percentages given are of individuals indicating they are White ethnicity; this included Irish Travelers. *Essex and RP are in bold because of their unique sociological histories; we did not include these data in our model.
